# The evolution of clot strength in critically ill COVID-19 patients: a prospective observational thromboelastography study

**DOI:** 10.1186/s12959-022-00365-3

**Published:** 2022-02-14

**Authors:** Syed Nabeel Muzaffar, Suhail Sarwar Siddiqui, Nilanchal Chakraborty, Afzal Azim

**Affiliations:** 1 Department of Critical Care Medicine, King George’s Medical University, 226003 Lucknow, Uttar Pradesh India; 2 Institute of Neurosciences, Kolkata, India; 3 Department of Critical Care Medicine, Sanjay Gandhi Postgraduate Institute of Medical Sciences (SGPGIMS), 226014 Lucknow, Uttar Pradesh India

**Keywords:** Thromboelastography (TEG), COVID-19, Hypercoagulability, fibrinolysis

## Abstract

The authors have done commendable work in exploring the utility of a comprehensive viscoelastic test for assessment of the coagulation cascade in Coronavirus disease 2019 (COVID-19) patients. This article published in your esteemed journal in November 2021 “The evolution of clot strength in critically-ill COVID-19 patients: a prospective observational thromboelastography study” found hypercoagulability in most of the patients at Intensive Care Unit (ICU) admission and also noted a persistently increased fibrin contribution to clot strength. However, we would like to comment upon a few points which may be of importance to the readers.

Dear Editor,

We read with great interest the article published by Neethling C et al in your esteemed journal in November 2021 issue, entitled “The evolution of clot strength in critically ill COVID-19 patients: a prospective observational thromboelastography study”. [1] The authors have done commendable work to explore the utility of thromboelastography, a comprehensive test for assessment of the coagulation cascade, in Coronavirus disease 2019 (COVID-19) patients with severe Acute Respiratory Distress syndrome (ARDS) requiring mechanical ventilation. In this study, hypercoagulability was found in the majority of patients at Intensive Care Unit (ICU) admission, which was also noted in a recent study by Fan BE et al [2]. These findings may be the reason behind microthrombosis, often seen in COVID-19 associated coagulopathy.[3]. In addition, it is worth appreciating that the authors have given importance to the assessment of clot stability by analyzing the fibrinolytic pathway, wherein a persistently increased fibrin contribution to clot strength was noticed. A similar concept of accentuated hypercoagulability secondary to fibrinolytic shutdown was highlighted by Coccheri S et al in 2020 [4]

However, a few comments which we would like to add are:

1. Study population:
  - It is difficult to explicitly differentiate whether the changes in clot strength were mediated by a cross talk between ARDS and coagulation, signifying an interplay between inflammation and coagulation, or by COVID severity per se. [5]
  - Most of the patients were obese (BMI > 30 Kg/m^2^), [6] which may itself contribute to hypercoagulability
  - Although patients with predisposition to thrombosis (like malignancy and pregnancy) were excluded from this study,analyzing how COVID-19 affects the changes in clot strength and composition in this subgroup may be an area of future research.
  - Influence of medications like antiplatelets and antifibrinolytics on TEG profile is not clear from the study.
  2. Methodology:
  - Baseline values of TEG may be different for different populations [7] and these values may also vary for different TEG machines [8] as has been seen in a recent study in COVID-19.
  - Disposal of COVID-19 positive blood and disinfection of TEG machine would also be prudent to know for the readers interested in replicating similar studies.
  3. Study findings:
  - Follow up period was for first 2 weeks only and the 14-30 day follow up period was missing in patients. It may be possible that the non-survivors had eventually progressed to a hypocoagulable TEG profile, [9] which has been associated with poor outcome in studies.
  - Only half of the patients (21/40) could be followed up serially over the 2 week observation period, which may not truly reflect dynamicity of changes in TEG parameters in survivors versus non-survivors in COVID patients and may require more studies in this area.

## Data Availability

The relevant references have been taken from either Pubmed or Google scholar. Doi links to the references have been attached.
